# The status of brown macroalgae *Fucus* spp. and its relation to environmental variation in the Finnish marine area, northern Baltic Sea

**DOI:** 10.1007/s13280-019-01175-0

**Published:** 2019-04-03

**Authors:** Henna Rinne, Sonja Salovius-Laurén

**Affiliations:** Faculty of Science and Engineering, Environmental and Marine Biology, Åbo Akademi University, Tykistökatu 6, 20520 Turku, Finland

**Keywords:** Distribution, Eutrophication, Indicator, Nitrogen, Phosphorus, Secchi depth

## Abstract

A large-scale loss of the habitat-forming macroalgae *Fucus vesiculosus* was reported around the Baltic Sea in the 1980’s, but only relatively local studies have reported its current status. We studied recent comprehensive mapping data in the Finnish marine area and compared reported *Fucus* occurrences in relation to its potential, to find out its current status on a larger scale. We also investigated the effects of water quality on *Fucus* occurrence and its depth penetration. Our results show that the status of *Fucus* is better in the Gulf of Bothnia than in the Gulf of Finland, both in terms of its occurrence rate and its depth distribution. Despite high potential in the outer Archipelago Sea, the status of *Fucus* is poor. The *Fucus* occurrence patterns are mainly related to salinity, exposure and Secchi depth, all positively affecting the *Fucus* occurrence rate and/or the lower limit of the *Fucus* zone.

## Introduction

The brown algae of the genus *Fucus* are widely distributed on the temperate rocky shores of the northern hemisphere, where they occur mainly in the intertidal zone (Graham and Wilcox 2003). In the brackish northern Baltic Sea, *Fucus* is the main genus of large canopy-forming macroalgae and it has an important role in providing shelter and food to many other species (Wikström and Kautsky 2007). Two species of *Fucus*, bladder wrack *Fucus vesiculosus* (L.) and narrow wrack *Fucus radicans* (Kautsky & Bergström) occur in the northern Baltic Sea (Bergström et al. 2005). *F. vesiculosus* is widely distributed extending to approximately 3.5 PSU salinity in the north (Bergström and Bergström 1999; Schagerström 2013) and to even lower salinities in the eastern Gulf of Finland (Lehvo and Bäck 2000; Ardehed et al. 2016). *Fucus radicans,* endemic to the Baltic Sea (Pereyra et al. 2009), occurs mainly in the Gulf of Bothnia (Johannesson et al. 2011; Rinne et al. 2018) but also along the west coast of Estonia and in the eastern Gulf of Finland (Pereyra et al. 2013; Schagerström 2013; Ardehed et al. 2016). The *Fucus* of the northern Baltic Sea show high genetic and morphological variation both on large and more local scales (Tatarenkov et al. 2007; Ardehed et al. 2016; Rinne et al. 2018), complicating their distinction to two species. Despite some ecological differences e.g. in their attractiveness to herbivores (Forslund et al. 2012; Gunnarsson and Berglund 2012), the species generally occupy the same ecological niche and their importance as habitat-formers and as food sources to many invertebrate herbivores is undisputable. The two species are hereafter referred to as *Fucus*.

The occurrence of *Fucus* in the Baltic Sea is strongly related to various environmental factors, with low salinity being the most important factor limiting its distribution (Bergström and Bergström 1999). Thus, its distribution range is expected to change with proceeding climate change that is expected to reduce the salinity of the northern Baltic Sea (Jonsson et al. 2018; Takolander et al. 2018). *Fucus* is more commonly found in exposed than sheltered areas (Rinne et al. 2011), and together with water transparency, exposure affects also the lower limit of *Fucus* occurrence (Eriksson and Bergström 2005; Rinne et al. 2011). Ice-scouring in winter often sets the upper limit of the *Fucus* zone in the northern Baltic Sea (Kiirikki 1996). Also biotic factors, such as competition for space with opportunistic algae during recruitment, epiphytic growth causing shading and restricting nutrient uptake, as well as meso-grazer herbivory play important roles in *Fucus* occurrence (Berger et al. 2004; Nilsson et al. 2004; Korpinen and Jormalainen 2008).

In the 1980’s, rapid declines in the occurrence of *Fucus* were reported in different areas of the Baltic Sea (Kangas et al. 1982; Pliǹski and Florczyk 1984; Rönnberg 1984; Rönnberg et al. 1985; Vogt and Schramm 1991). The decline was linked to various eutrophication effects, such as higher water turbidity and increase in opportunistic algae (Kangas et al. 1982; Rönnberg et al. 1985; Vogt and Schramm 1991), but also to a mass occurrence of the isopod *Idotea balthica* (L.) that grazes on *Fucus* (Kangas et al. 1982). The observed declines in *Fucus* occurrence coinciding with proceeding eutrophication, and the negative responses of the species to both direct and indirect effects of eutrophication in laboratory experiments (Bergström et al. 2003; Berger et al. 2003) strongly indicate that the species is sensitive to eutrophication related effects, although *Fucus* may in some areas thrive in relatively high nutrient concentrations (Rinne et al. 2011). Also the depth penetration of *Fucus* decreases with proceeding eutrophication (Kautsky et al. 1986; Eriksson et al. 1998; Torn et al. 2006), most likely as a response to lower water transparency (Torn et al. 2006) and higher sedimentation rates (Eriksson and Johansson 2005). Based on this, the maximum depth of the continuous *Fucus* belt is measured as part of the monitoring related to the Water Framework Directive (WFD) in Finland (Ruuskanen 2016).

Despite more local-scale studies on the status of *Fucus* (Snickars et al. 2014; Vahteri and Vuorinen 2016) the general occurrence patterns of *Fucus* on a larger scale in the northern Baltic Sea remain undescribed, including its relationship to environmental variability. Furthermore, there is little knowledge on the spatial variation in the depth penetration of *Fucus* on a larger scale, despite its use in monitoring. To gain a holistic view on the current occurrence patterns and status of *Fucus* in the Finnish marine area, and to evaluate its coupling to the variation in the environment, we studied the current presence of *Fucus* in relation to its potential in water bodies defined in the Water Framework Directive. Furthermore, we investigated spatial variation in the lower limit of *Fucus* belt across the Finnish marine area and its relation to environmental variability.

## Materials and methods

### Study area

**Fig. 1:**
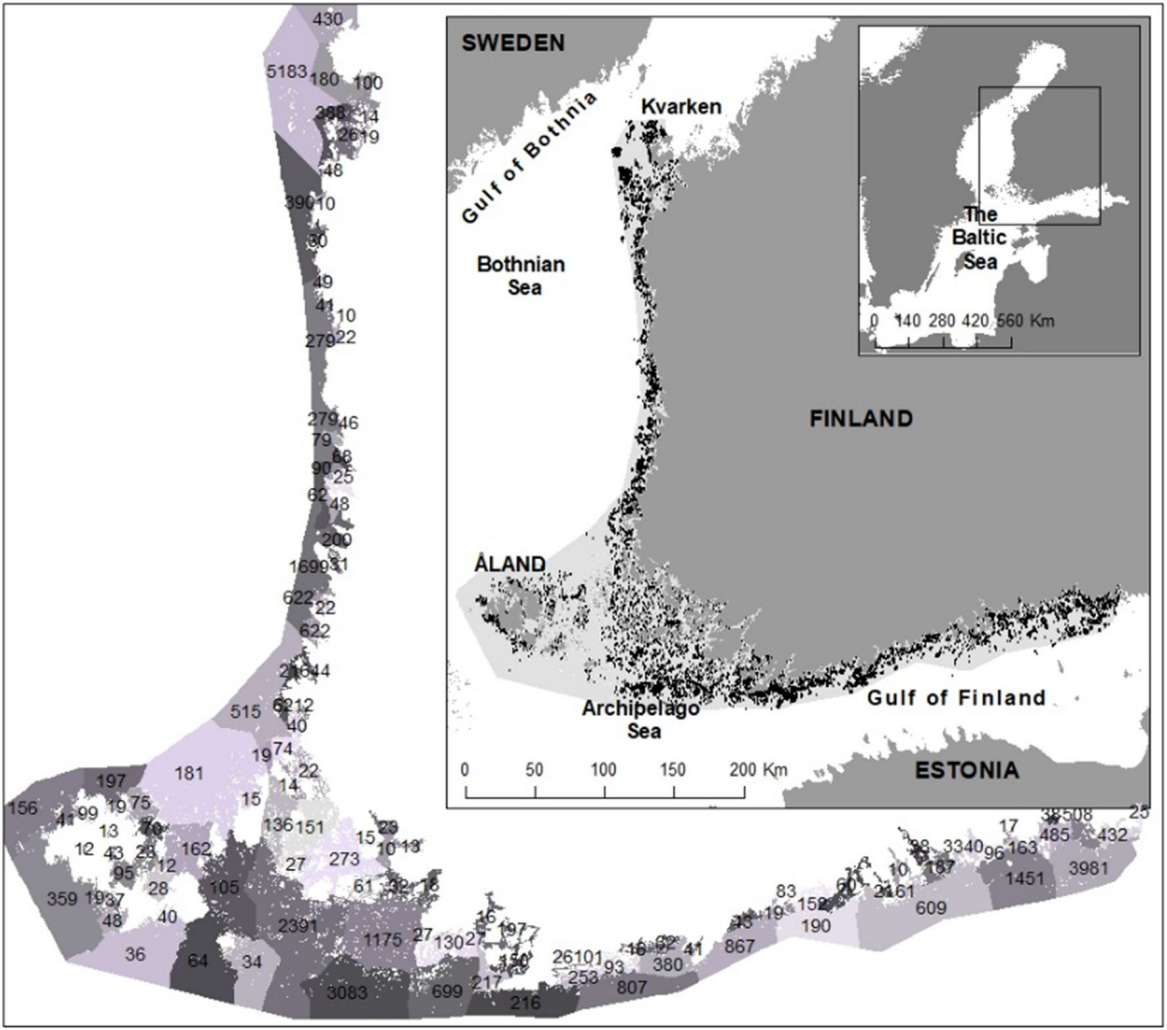
The study area in the northern Baltic Sea, showing all surveyed sites potential for *Fucus* occurrence with black dots (37 469 sites). The largest map shows the division into WFD areas (colouring random and only to visualize the WFD areas), and the number of surveyed sites potential for *Fucus* within the 126 WFD areas included in the study

The study area is situated in the northern Baltic Sea, and covers the distribution area of *Fucus* in the coastal waters of Finland (Fig. 1) (Nielsen et al. 1995; Bergström and Bergström 1999). The study area is characterized by a distinct salinity gradient (2–6.5 PSU), with salinity decreasing northward in the Gulf of Bothnia and towards east in the Gulf of Finland. In the area, archipelagos act as transition zones between the coast and the open sea, forming mainland to open sea gradients in exposure, salinity and water quality, all generally increasing towards the open sea.

### Fucus data

We used an extensive macrophyte dataset collected during the Finnish national marine mapping programme VELMU (2004–2016) and data from the Husö Biological Station, Åbo Akademi University, in Åland (2004–2017), that together cover the whole Finnish marine area. Altogether, the data consisted of over 116 000 survey points, gathered using drop-video surveys (99 730) and SCUBA-diving transects (16 330 points along transects). All surveys were carried out in the summer time (June-October).

During VELMU surveys, the SCUBA-diving transects are placed using stratified random sampling design, to ensure the coverage of different environmental gradients (e.g. exposure, different substrates) (Anonymous 2015). At each site, the transect (100 m) is placed perpendicular to the shoreline. In all transects, recordings of species cover and substrate cover (%) are made from an area of 4 m^2^ (hereafter “study points”) either at 10 m horizontal (along the seafloor) or 1 m vertical (in the water column) intervals along the transects to get reliable data from the entire transect. The species-specific cover (%) of macrophytes, macroalgae and sessile fauna, as well as substrate, is recorded on a continuous scale from 0 to 100%. The substrate recordings are based on a 11-level classification (bedrock, boulders > 300 cm, boulders 120–300 cm, boulders 60–120 cm, stones 10–60 cm, stones 6–10 cm, gravel, sand, silt, clay and mud), modified from Wentworth (1922). Also the depth of the study point is recorded. If vegetation ends before 100 m is reached, (e.g. steep transects), the final transect surveyed may be shorter. In Åland, most of the dive transects (except for data collected in 2017) were only 50 m long and the species cover was recorded from a smaller area (0.5–2 m^2^).

The drop-video surveys are planned using stratified random sampling design (stratification according to depth, exposure, turbidity and salinity) but also grid sampling with 100 m intervals has been applied within some protected areas (Anonymous 2015). The drop-video surveys are carried out from a boat, where a video-recorder equipped with lights is lowered with a cable near the seafloor and the seafloor is filmed for one minute (covering 20 m^2^ on average). The videos are later analysed to record substrate and species cover (%) (Anonymous 2015).

### The presence of Fucus in relation to its potential

As *Fucus* individuals are observed reliably using both SCUBA-diving and drop-video, we used survey data gathered using both methods when evaluating the presence of *Fucus* in relation to its potential occurrence. The potential sites for *Fucus* occurrence were defined as surveyed sites with ≥ 10% hard substrate (rock, boulders, stones or gravel) in areas with adequate light levels for *Fucus* growth. To define the areas with sufficient light levels for *Fucus*, the photic zone (1% PAR at surface left) was first calculated using a Secchi depth layer based on satellite data (see chapter on environmental variables) and conversion coefficients of the quartile method presented in Luhtala and Tolvanen (2013). To obtain light levels adequate for *Fucus* growth, the photic zone was further multiplied with 0.6 (representing 60% of the total photic depth). This coefficient was based on Rohde et al. (2008) where 1% PAR was reached at 10.7 m and the physiological limit to *Fucus* growth was determined to be at 6 m depth. The resulting layer was further intersected with a depth model (available at: http://paikkatieto.ymparisto.fi/VELMU_karttapalvelu with metadata) to find seafloor areas where light levels are adequate for *Fucus* growth. The suitability of the produced layer was checked by intersecting the *Fucus* observations with the layer. Only 1.6% of 11 551 *Fucus* observations were found outside the area here defined suitable for *Fucus* growth. Although there were some observations outside the area, the layer was found adequate, as we rather underestimated the potential sites for *Fucus* than overestimated them.

The produced layer describing areas with light levels suitable for *Fucus* growth was intersected with all surveyed sites with ≥ 10% hard substrate. Altogether 37 469 of the total number of 116 000 survey points were defined as potential sites for *Fucus* occurrence (Fig. 1). To describe the potential occurrence of *Fucus* in relation to its actual presence geographically, we used a division into water bodies used in the implementation of the WFD (hereafter referred to as WFD areas) in Finland (Fig. 1). The potential presence of *Fucus* in relation to its actual occurrence within each WFD area (= *Fucus* occurrence rate) was calculated as

$$ \left( {{\text{Number}}\,{\text{of}}\,Fucus\,{\text{observations}}\,{\text{in}}\,{\text{a}}\,{\text{WFD}}\,{\text{area}}/{\text{number}}\,{\text{of}}\,{\text{surveyed}}\,{\text{potential}}\,{\text{sites}}\,{\text{for}}\,Fucus\,{\text{occurrence}}\,{\text{in}}\,{\text{a}}\,{\text{WFD}}\,{\text{area}}} \right)\,*\, 100 $$

If a WFD area had less than 10 potential *Fucus* sites, the area was not included in the study, because the surveys were not exhaustive enough to evaluate potential occurrence rates. The final dataset included 37 263 surveyed potential occurrence sites in 126 WFD areas.

### The lower limit of the Fucus zone

Only SCUBA-diving transect data were used when evaluating the lower limit of the *Fucus* zone. The *Fucus* zone was defined as at least two subsequent ≥ 10% covers of *Fucus* on a transect and the lower limit was set as the deepest occurrence of ≥ 10% cover. There were altogether 328 transects with *Fucus* zones. Each transect was checked for the continuation of hard substrate below the *Fucus* zone, to make sure that the lower limit was set due to other factors than unsuitable substrate, and that the transect continued below the *Fucus* belt. Only 186 transects remained after checking for substrate limitation, thus almost half of the sites with *Fucus* zones were unsuitable for assessing the lower limit of *Fucus*.

A comparison to earlier data on *Fucus* depth distribution was also made, where data were available. In the earlier literature, there were often no actual recordings of the lower limit of the *Fucus* zone, but instead, the depth distribution ranges of *Fucus* were recorded (e.g. occurred at depths of 0.5–7 m). In those cases, we used the lowest depth recorded to resemble the past lower limit of the *Fucus* zone. The spatial matches to our data were made based on maps or site names from the studies.

### Environmental variables

To relate the environmental variability to *Fucus* occurrence rate and to the lower limit of the *Fucus* zone, we used environmental data layers on Secchi depth, total phosphorus and nitrogen concentrations (P-tot and N-tot respectively), surface salinity and exposure (100 m × 100 m grids for N-tot and P-tot, 300 m × 300 m grid for Secchi depth, 20 m × 20 m grid for salinity and 25 m × 25 m grid for exposure). The N-tot and P-tot grids are interpolations (produced using Spline with barriers function in ArcGIS 10.5) on data obtained from the national water quality database Hertta (available from http://www.syke.fi/avointieto) as well as from the Government of Åland and Husö Biological Station, Åbo Akademi University. Average summer (June–August) values 2004–2015 from 1 to 10 m depth were used in the interpolations. The surface salinity layer is a result of a model based on monitoring data from June to August 2004 to 2015 (data sources the same as for nutrient data) that also takes into account the effects of rivers. The Secchi depth layer was calculated based on satellite censor images (MERIS) from June to August in 2003 to 2011 (both salinity and Secchi layers available at http://paikkatieto.ymparisto.fi/VELMU_karttapalvelu with metadata). We found these environmental data layers based on long-term averages suitable for the study due to correspondence in timing with the vegetation surveys, and also because we wanted to relate the *Fucus* parameters to the general state of the marine area, not to a specific point in time. For exposure, we used a wave exposure index grid covering the Finnish territorial waters, calculated using the simplified wave exposure model (SWM) (Isæus 2004). To obtain the environmental variable values for each macroalgal transect (to relate them to the lower limit of the *Fucus* zone), we used “Extract values to point” tool in ArcGIS 10.5. To get mean values of environmental parameters for each WFD area (to relate them to the *Fucus* occurrence rate within the WFD), we used “Zonal statistics as a table” tool in ArcGIS (WFD areas as zones), which calculated the mean of the pixel values within each WFD area.

### Data analysis

The effects of environmental variables on the occurrence rate of *Fucus* were explored with N-tot, P-tot, Secchi depth, salinity and exposure (mean/WFD area) and their two-way interactions as explanatory variables and the occurrence rate of *Fucus* within a WFD area as the response variable. Before the analysis, the correlations between the environmental variables were checked. Furthermore, as the mean amount of hard substrate varied to some extent across the WFD areas included in the study (i.e. areas with > 10 potential sites for *Fucus*), its correlation to *Fucus* occurrence rate was checked and found low (*r* = 0.12). Therefore it was not included in the model as an explanatory variable. Since the *Fucus* occurrence data were zero-inflated (20 out of 126 WFD areas had no *Fucus*), we applied a hurdle model to the data. Hurdle model is a two-step model that consists of a presence/absence model (all values > 0 converted to 1) and a truncated positive model that includes only values > 0 (Zuur and Ieno 2016). The two models can be merged for prediction by multiplying the predictions of the models. The positive model was run using beta regression, suitable for proportional data, using glmmADMB library in R (Fournier et al. 2012; Skaug et al. 2016). Non-significant interactions were dropped from the final model. The binomial model was run with the glm function in R (R Core Team 2017), including only the interaction term that was found significant in the positive model, to allow model comparison. The relationship between environmental variables and the lower limit of *Fucus* was explored using a general linear model, with the log transformed lower limit (in metres) as the response variable and N-tot, Secchi depth, salinity and exposure as explanatory variables, including their two-way interactions. P-tot was dropped from the analysis, due to high correlation with N-tot (0.84, Table 1). In both analyses, the model residuals were checked for normality and homoscedasticity.

**Table 1:** The Pearson correlations between environmental variables. The values in the lower left corner are the correlation coefficients between mean values/WFD areas (used when studying the relationship between the occurrence rates/WFD area and the environment). The values in the upper right corner are the correlation coefficients (*in italics)* between values at dive transects (used when studying the relationship between the lower limit of occurrence and the environment). The bold values refer to statistical significance (p < 0.05)

	Salinity	Exposure	Secchi depth	N-tot	P-tot
Salinity		*0.07*	***0.64***	**− *0.75***	**− *0.76***
Exposure	**0.18**		*0.08*	**− *0.44***	**− *0.36***
Secchi depth	**0.70**	**0.36**		**− *0.46***	**− *0.58***
N-tot	**− 0.44**	**− 0.44**	**− 0.45**		***0.84***
P-tot	**− 0.38**	**− 0.27**	**− 0.42**	**0.67**	

## Results

### The presence of *Fucus* in relation to its potential

**Fig. 2:**
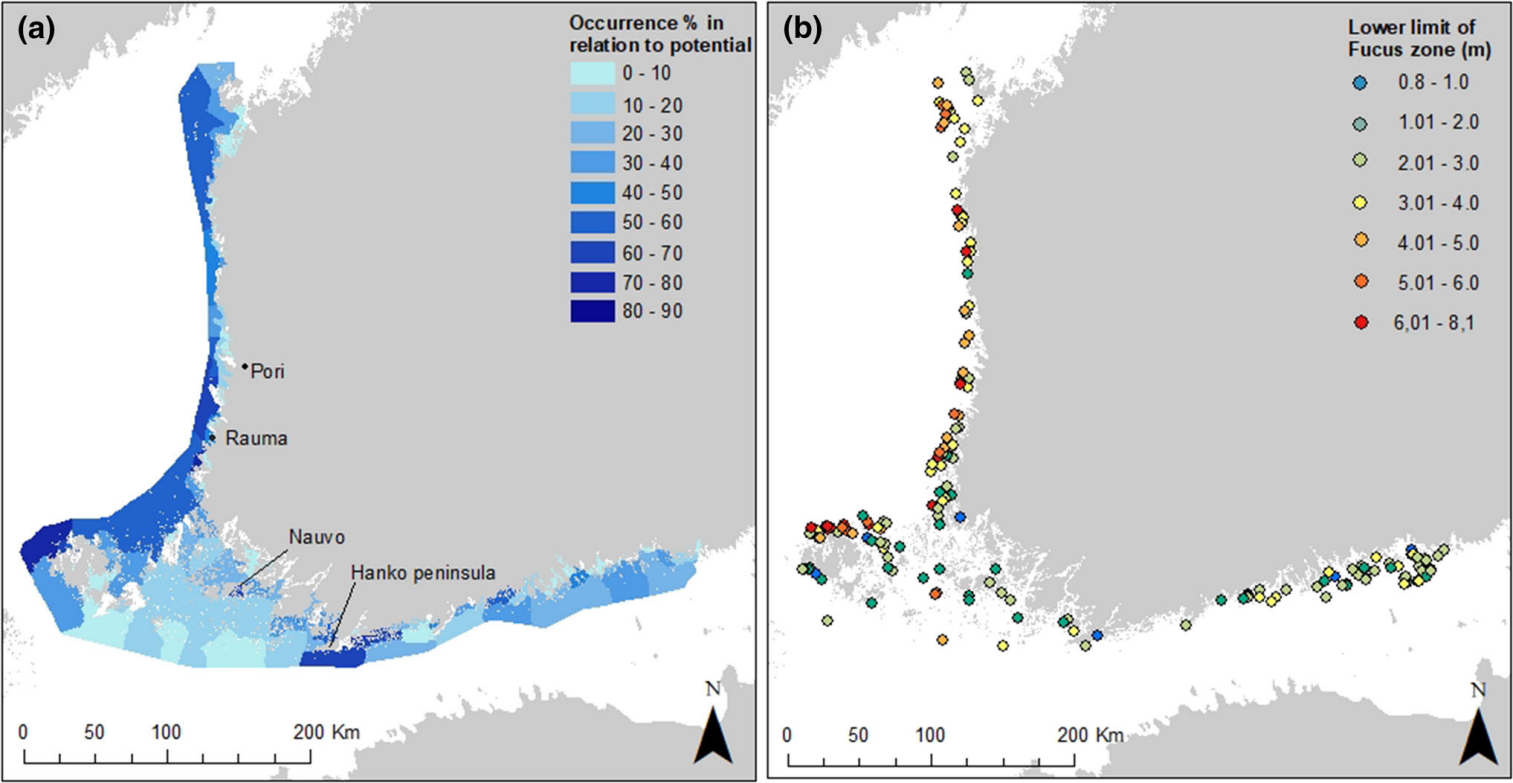
**a** The presence of *Fucus* in relation to its occurrence potential within the 126 WFD areas that had > 10 surveyed potential sites for *Fucus* and **b** the lower limit of *Fucus* zone (m) in the study area (186 sites)

The occurrence of *Fucus* in relation to its potential was higher along the western coast than in the Gulf of Finland, with the exception of western Gulf of Finland where areas to the south and west of Hanko peninsula reached relatively high occurrence rates (approximately 70%, Fig. 2a). Throughout the study area, the WFD areas close to the mainland had little *Fucus* (0-20%), especially in the eastern Gulf of Finland, in the northern Bothnian Sea and in Kvarken. The innermost parts of the Archipelago Sea were not included in this study, as very few surveyed sites were potential for *Fucus* growth due to low existence of photic hard bottom in the area. In the middle archipelago (in the Archipelago Sea), *Fucus* was found at 20–30% of potential sites, except for one relatively sheltered WFD area between larger islands (Lillandet and Storlandet in Nauvo) that had a 67% occurrence rate. Only 0–20% of the potential sites contained *Fucus* in the outer Archipelago Sea. The highest occurrence rates were reached northwest of Åland Islands, where up to 75% of potential sites contained *Fucus*, and also in the area south of the city of Rauma (74%).

### The lower limit of the *Fucus* zone

In general, the *Fucus* zone penetrated deeper in the Bothnian Sea and in Kvarken, than in the Gulf of Finland (Fig. 2b). Especially the more exposed sea areas along the west coast had deeper occurrences of *Fucus* zones than the areas close to the mainland. E.g. in Kvarken outer archipelago, the *Fucus* zones reached down to 4–6 m, while in the inner parts they reached only 3–4 m depths. Further south, the deep-penetrating *Fucus* zones were most commonly found in northern Åland (6–7 m, even down to 8 m). In the eastern Gulf of Finland, the lower limit of the *Fucus* zone was mainly between 1 and 3 m, although at some sites the zone reached 3–4 m depth. Lower limits of 1–3 m were generally found in the Archipelago Sea, and in the eastern and southern parts of Åland Islands. The few sites in the outer Archipelago Sea that had *Fucus* zones, they reached down to 4–5 m, or even to 6 m.

A comparison to historical data (Table 2) revealed a drastic decline in the lower limit of the *Fucus* zone over decades in all areas, except for western Åland (although the comparative data from western Åland were more recent than in some other areas). In the central and eastern Gulf of Finland, there was only a slight decrease in depth penetration from the early 1990’s.

**Table 2:** A comparison between former recordings of the lower limit of *Fucus* zone in the Finnish marine area and the ones found in this study. Also maximum depth of *Fucus* (≥ 10% coverage) recorded at all studied sites (including video points) within the area is given in the last column

Area	Reference (year of data collection after reference)	Former lower limit of the *Fucus* zone	Current lower limit of the *Fucus* zone (recorded on transects)	Max depth recorded with ≥ 10% *Fucus* coverage (all data)
Southern Bothnian Sea, Uusikaupunki archipelago	Häyrén (1950)	7 m**	2.5 m (1 transect close to Häyrén’s study area)	2.5 m
Western Åland	Rönnberg (1984), 1982	7 m (exposed)** 3 m (sheltered)**	3.1–7 m, max 7.5 m (exposed) 3 m (sheltered)	10.6 m
South-eastern Åland, Kökar	Andersson (1955)	11 m**	1.1 m (only one transect in the area)	2.6 m
Archipelago Sea, middle archipelago	Ravanko (1968)*	9 m**	2–3 m, max 5 m	5 m
Archipelago Sea, Seili area	Haahtela and Lehto (1982), 1969	3 m**	1.4–1.8 m (2 transects close to Seili)	1.9 m
Western Gulf of Finland, Tvärminne	Purasjoki (1936), Strandström (1980) and Bäck and Ruuskanen (2000), 1991	7–8 m, max 10 m** 8 m** 5 m (exposed), 4.5 m (sheltered)	0.9–2.4 m (2 transects in the area)	4.3 m
Helsinki, Central Gulf of Finland	Bäck and Ruuskanen (2000), 1991	5.5 m (exposed)	1.5–3.8 m (5 transects, closer to mainland, yet exposed)	4.4 m
Kotka, Eastern Gulf of Finland	Bäck and Ruuskanen (2000), 1991	3 m (exposed)	1.8 m (same island as before)	3.1 m
Virolahti, Eastern Gulf of Finland	Bäck and Ruuskanen (2000), 1991	3 m (sheltered)	2.2–2.9 m (3 transects in the area)	5.5 m

*General description covering a large area in the Archipelago Sea

** Based on a description of the depth zone where *Fucus* generally occurs, does not necessarily correspond to the lower limit of *Fucus* as defined in this study (≥ 10% coverage)

### Variation in the environment

**Fig. 3:**
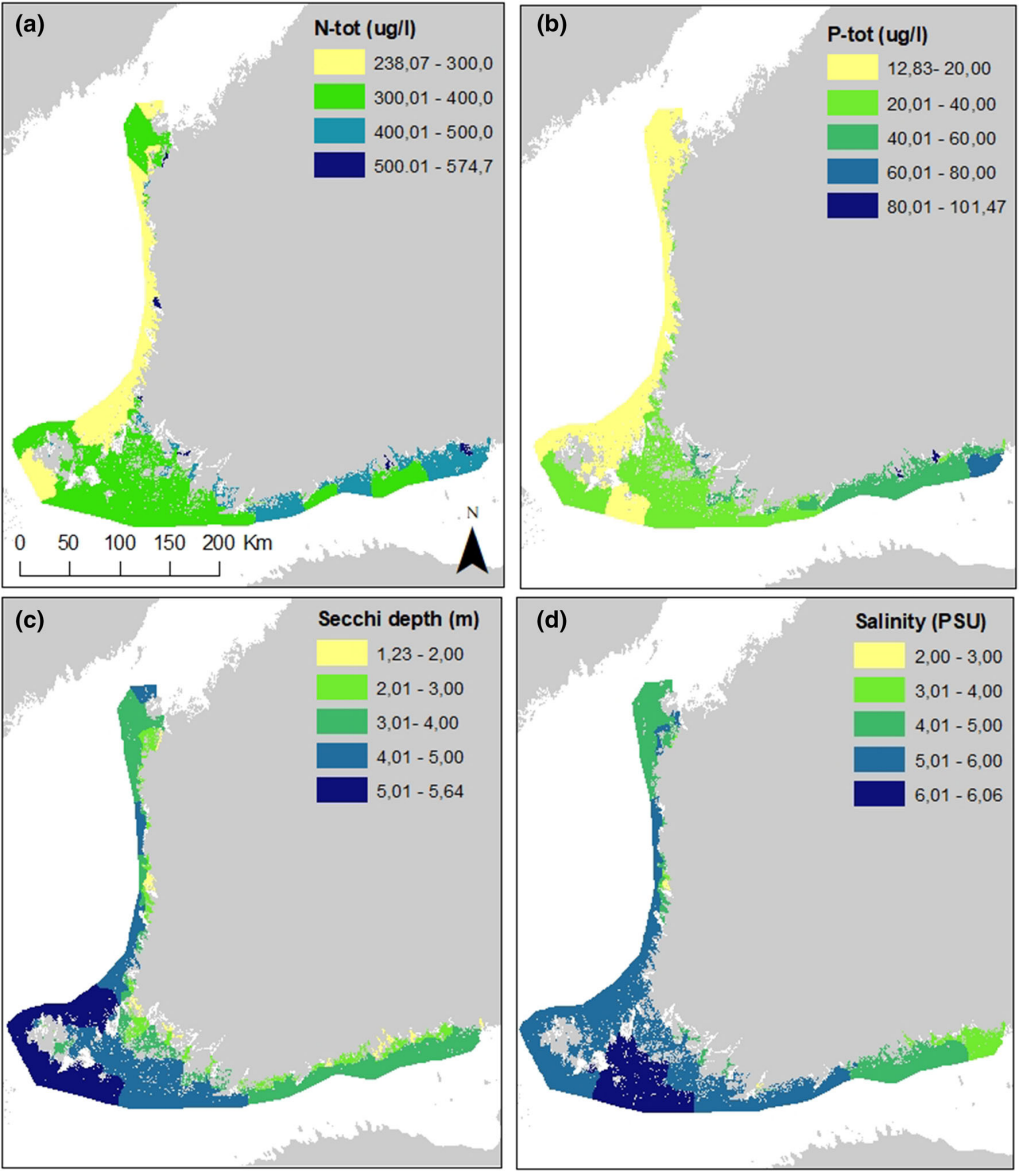
The mean/WFD area for** a** N-tot,** b** P-tot,** c** Secchi depth and** d** salinity across the WFD areas included in the study

Total nitrogen and phosphorus concentrations were clearly higher in the WFD areas of the Gulf of Finland than in the Gulf of Bothnia (Fig. 3). The peak values for both nutrients were reached in the inner archipelago areas in the eastern Gulf of Finland, and for nitrogen also close to large river mouths in the Archipelago Sea and in the Gulf of Bothnia. The WFD areas in the Archipelago Sea and Åland islands showed intermediate levels of both nutrients. The Secchi depth was highest around Åland islands and lowest in the sheltered areas close to the mainland. Salinity was highest in the outer parts of the Archipelago Sea and decreased generally towards east and north. Also the inner archipelago areas with high freshwater input had relatively low salinities. The correlations between environmental variables are presented in Table 1.

### The relationship between environmental variables and Fucus occurrence

The beta regression model with *Fucus* occurrence/WFD area as a response variable revealed that *Fucus* occurrence rate in WFD areas increased with increasing salinity, and that also Secchi depth had positive effects (Table 3). There was a significant interaction between salinity and Secchi depth, indicating that the positive effects of Secchi depth are more profound in lower salinities. Exposure had positive effects on the presence of *Fucus* (binomial model). No significant relationships between nutrient concentrations (N-tot and P-tot) and *Fucus* occurrence rate were found.

**Table 3:** The results of the hurdle model where the effects of environmental variables (salinity, exposure, Secchi depth, N-tot and P-tot) on the occurrence rate of *Fucus* were modelled in a two-step model: a truncated beta regression model for occurrence rate values > 0 and a binomial model for presence/absence. Significance levels are indicated by asterisks: ***p < 0.001, *ns* not significant

	Positive truncated model (*n *= 106)			Binomial model (*n* = 126)		
	Estimate	Std error	z value	Estimate	Std error	z value
Salinity	1.51	0.42	3.62***	1.37	0.97	1.41^ns^
log Exposure	0.04	0.06	0.64^ns^	0.92	0.32	2.83***
Secchi depth	2.48	0.73	3.41***	3.81	2.39	1.59^ns^
N-tot	0.002	0.002	0.92^ns^	− 0.004	0.006	0.65^ns^
P-tot	− 0.004	0.007	0.23^ns^	0.02	0.03	0.92^ns^
Salinity * Secchi	− 0.45	0.13	− 3.43***	− 0.60	0.42	− 1.42^ns^

The variation in the lower limit of the *Fucus* zone was strongly related to exposure and Secchi depth (Table 4), with deeper reaching zones in the more exposed areas and in higher Secchi depths. Also salinity had a positive effect on the depth penetration of *Fucus*. The significant negative interaction between salinity and Secchi depth indicated stronger positive effects of Secchi in lower salinities. Furthermore, nitrogen concentration had a weak positive effect on the lower limit of *Fucus*. The significant negative interaction between exposure and nitrogen concentration indicated weaker positive effects of exposure in higher nitrogen concentrations.

**Table 4:** The effects of environmental variables (salinity, exposure, Secchi depth and N-tot) on the lower limit of *Fucus* zone, modelled using a general linear model. Significance levels are indicated by asterisks: *** *p* < 0.001, **p < 0.01 *p < 0.05

	Estimate	Std error	t-value
Salinity	0.66	0.21	3.21**
Log exposure	0.27	0.07	3.84***
Secchi depth	1.19	0.31	3.87***
N-tot	0.005	0.002	2.29*
Log exposure * N-tot	− 0.0005	0.0002	− 2.42*
Salinity * Secchi	− 0.21	0.06	− 3.68***

## Discussion

### Fucus occurrence and its coupling to environmental parameters

*Fucus* occurred most frequently in northern Åland, an exposed area with relatively high salinity, high Secchi depth and relatively low nutrient concentrations. The areas with high occurrence rates of *Fucus* formed a continuum in the more open sea from northern Åland northwards, all the way to the Kvarken area, with only a slight decrease in the occurrence rate near Pori (Fig. 2a) where the large river Kokemäenjoki with yearly mean flow of 245 m^3^ s^−1^ (Verta and Triipponen 2011) discharges into the Bothnian Sea. Based on earlier surveys, the northern Åland and the Bothnian Sea have not experienced large-scale losses of *Fucus* (Rönnberg 1984; Pogreboff and Rönnberg 1987).

Despite the higher *Fucus* occurrence rate in the Bothnian Sea than in the eutrophied Gulf of Finland, we found no direct coupling to nutrient concentrations. This is likely due to intermediate (30–40%) occurrence rates of *Fucus* in the open sea areas of the eutrophied eastern Gulf of Finland, and that the occurrence rates in the Gulf of Finland showed no general increase towards the less eutrophied areas. Both salinity and Secchi depth had significant positive effects on *Fucus* occurrence rate, most likely influenced by the generally higher occurrence rates in the outer archipelago (with higher Secchi depths and salinity) than in the inner archipelago. The weaker effects of Secchi depth in higher salinities (indicated by the significant negative interaction) could at least partly be due to the low occurrence rates in the outer Archipelago Sea, the area with the highest salinity and high Secchi depths. The changing effects of Secchi depth in different salinities could also indicate that Secchi depth as a measure of light attenuation could differ between areas, due to spatial variation both in factors contributing to Secchi depth (Harvey et al. 2019) and in correlations with other environmental factors (Snickars et al. 2014).

On a larger geographical scale, the occurrence rate did not follow the decreasing salinity gradient towards the north and east as the area with the highest salinity, the outer Archipelago Sea, had very low occurrence rates. However, in the adjacent areas, (western Gulf of Finland, western parts of Åland islands and southern Bothnian Sea) the occurrence rates were among the highest, strongly emphasizing lower than expected occurrence rates in the outer Archipelago Sea.

In the Archipelago Sea the most severe losses of *Fucus* in the 1980’s were reported in the middle archipelago (Mäkinen et al. 1984; Rönnberg et al. 1985). Although some improvement in *Fucus* occurrence has been reported at sheltered sites (Vahteri and Vuorinen 2016), the area shows mainly only 20–30% occurrence rates (despite the one sheltered WFD area with 67% occurrence rate). The exceptionally low occurrence in the outer Archipelago Sea coincides with recent findings based on more limited data (Snickars et al. 2014; Vahteri and Vuorinen 2016). The steepest decrease in the outer Archipelago Sea seems to have happened after mid 1990’s (Vahteri and Vuorinen 2016), suggesting a “wave” of *Fucus* loss proceeding from middle to outer archipelago. The continued eutrophication development of the area has been suggested as the main factor contributing to the low occurrence of *Fucus* in the outer archipelago (Snickars et al. 2014; Vahteri and Vuorinen 2016). However, the frequent occurrences in the more eutrophied areas do not support this conclusion (see also Rinne et al. 2011). It is also possible that some parts of the outer Archipelago Sea are too exposed to *Fucus* (Jonsson et al. 2006) although there are areas with comparable exposure showing high occurrence rates in the open sea areas of the Bothnian Sea and the Gulf of Finland. Furthermore, earlier studies (Rönnberg et al. 1985; Vahteri and Vuorinen 2016) reported *Fucus* zones in the outer Archipelago Sea, confirming that *Fucus* has occurred in the area. Also ice-scouring likely influences *Fucus* occurrence in the outer Archipelago Sea, especially with *Fucus* occurring in shallower water than before, but one would expect higher impact of ice in the areas where ice is more persistent, e.g. in the eastern Gulf of Finland and in the Bothnian Sea. High grazing pressure in the Archipelago Sea could partly explain the observed pattern as declines in *Fucus* have been reported to coincide with mass occurrences of *Idotea* (Haahtela and Lehto 1982; Kangas et al. 1982). Although herbivory is expected to play a smaller role in more exposed areas (Menge and Sutherland 1987), in the south-eastern coast of Sweden the grazing of *I. balthica* was considered the main factor contributing to a decrease in *Fucus* abundance in an exposed area (Nilsson et al. 2004). Despite little data on herbivore abundances across larger spatial scales (but see Viitasalo et al. 2017 for data on *I. balthica* presence), it is likely that herbivore abundances in the Archipelago Sea are higher than in areas with lower salinities, as the low-salinity environments are physiologically more challenging for the marine *I. balthica* (Leidenberger et al. 2012). This theory is further supported by the fact that no large-scale losses of *Fucus* have been reported in the eastern Gulf of Finland or in the Bothnian Sea. In the eutrophied conditions, the (herbivory-induced) loss of *Fucus* could lead to a situation, where *Fucus* is unable to recolonize the area due to high competition with filamentous algae (Berger et al. 2003). This may be the current situation in the Archipelago Sea. Furthermore, the higher nutrient conditions improve the quality of both filamentous algae and *Fucus* as food sources for herbivores, further enhancing herbivore growth and reproduction (Hemmi and Jormalainen 2002) and leading to higher herbivore abundances. However, the reasons behind the clear differences in *Fucus* occurrence rates between the Archipelago Sea and the adjacent areas, the western Gulf of Finland and Åland, need further investigations.

In contrast to the exposed outer Archipelago Sea, the effects of eutrophication on *Fucus* occurrence in the innermost Archipelago Sea are undisputable. The species has disappeared from the innermost archipelago (earlier surveys in the area by e.g. Peussa and Ravanko 1975). Although surveys within the mapping programme extended to the area, only few sites suitable for *Fucus* growth were found (often < 10 sites/WFD area) and the innermost areas were therefore excluded from this study. Due to eutrophication, turbid waters and high sedimentation, the area has largely lost its potential to host *Fucus,* as very little suitable photic hard substrate remains.

### Lower limit of the Fucus zone and its coupling to environmental parameters

The lower limit of the *Fucus* zone showed the same general pattern as the occurrence rate: the deepest penetrating zones were found in the northern Åland islands and in the more open sea areas of the Gulf of Bothnia. Relatively little information on the depth distribution of *Fucus* from the earlier decades exists, but where data were available, mainly drastic declines in the lower limit of the *Fucus* zones were found. Although none of the transects included in our study were on exact same locations as in earlier studies, and sometimes the study area descriptions in former studies were relatively unspecific, we believe that the comparison done gives a general idea on the magnitude of decline in depth penetration that has happened in the Archipelago Sea and the adjacent areas. Of the southern parts of the Finnish marine area, only *Fucus* in western Åland seem to be doing well, also concerning depth distribution. A more recent decline in *Fucus* depth penetration has been recorded during macroalgal monitoring in the Gulf of Finland (Ruuskanen 2016), where the lower limit of the *Fucus* zone has moved upwards since the early 2000’s, more drastically in the western Gulf than in the east.

The currently observed variation in the maximum depth of the *Fucus* zone was coupled with variation in Secchi depth. This supports earlier studies where positive relationships between water transparency and depth penetration of *Fucus* have been found in the central and northern Baltic Sea (e.g. Eriksson and Bergström 2005; Torn et al. 2006; Rinne et al. 2011). The lower limit of *Fucus* zone followed both the inner-outer archipelago gradient, with generally deeper reaching zones in the more open sea areas, and the variation in Secchi depth on a larger geographical scale. The strong effects of exposure on the lower limit of the *Fucus* zone (Rinne et al. 2011), not only on the upper limit (Kiirikki 1996; Eriksson and Bergström 2005), were also confirmed in this study. This supports the use of differing thresholds for status classes in the sheltered and the exposed areas, when the lower limit of the *Fucus* zone is used in the status assessments (Aroviita et al. 2012). The interaction between nitrogen concentration and exposure, suggests that the positive effects of exposure on the depth penetrations are not as strong in higher nitrogen concentrations. The positive, rather than negative effects of nitrogen concentrations on the lower limit of the *Fucus* zone, suggest that higher nutrient concentrations do not have direct negative effects on the depth penetration of *Fucus* (see also Rinne et al. 2011), but instead, the eutrophication effects on *Fucus* depth penetration are expressed more indirectly via increased sedimentation and decreased water transparency (Eriksson and Bergström 2005; Rinne et al. 2011).

## Conclusions

We found higher occurrence rates and deeper reaching *Fucus* zones in northern Åland and in the Bothnian Sea in comparison to the Gulf of Finland and the Archipelago Sea, suggesting a relatively good status of the species in the Bothnian Sea. However, the lower limits of *Fucus* are today considerably closer to the surface in all sea areas (except northern Åland Islands) compared with earlier studies. Both the occurrence rate and the lower limit of *Fucus* zone were related to Secchi depth, suggesting that water transparency is a key environmental factor affecting the status of the species. Salinity affected both the occurrence rate and the lower limit of the *Fucus* belt, and it seemed that in higher salinities, the positive effects of Secchi depth on both variables were not that clear. The relationship between the lower limit of the *Fucus* zone and Secchi depth suggests that the use of the lower limit of the *Fucus* zone as an indicator of the status of the northern Baltic Sea is justified, when the effects of exposure and salinity are accounted for in the assessments.

In general, the current bad status of *Fucus* in the Archipelago Sea is alarming, as with the future climate change the area is expected to be the key area for *Fucus* distribution within the Finnish marine area (Jonsson et al. 2018). The role of herbivory in shaping *Fucus* occurrence patterns in the area and also on a larger scale needs further studies. While the reasons behind the low occurrence in the outer Archipelago Sea remain unclear, the observed changes in depth penetration are likely due to reduced water transparency. Furthermore, the inner Archipelago Sea has to a large extent lost its potential to host *Fucus,* as very little suitable hard substrate remains in the area. Therefore, it is clear that human induced changes have played a significant role in the *Fucus* occurrence patterns that we observe today.
